# Retraction: 2-Methoxyestradiol alleviates neuroinflammation and brain edema in early brain injury after subarachnoid hemorrhage in rats

**DOI:** 10.3389/fncel.2026.1860637

**Published:** 2026-04-27

**Authors:** Frontiers Editorial Office

The journal retracts the article cited above.

Following publication, concerns were raised regarding the integrity of the images in the published figures: image duplication concerns were identified in Figure 2, with images having appeared in a previously published article.

The authors and their institution have remained unresponsive during the investigation, which was conducted in accordance with Frontiers' policies. As a result, the data and conclusions of the article have been deemed unreliable and the article has been retracted.

This retraction was approved by the Chief Editors of Cellular Neuroscience and the Chief Executive Editor of Frontiers. The authors received a communication regarding the retraction and had a chance to respond. This communication is recorded by the publisher.

Frontiers would like to thank the Publishing Ethics Team at Elsevier and users of PubPeer for alerting us to this issue.

